# Supplementary figures and images for: Nckx30c, a Drosophila K+-dependent Na+/Ca2+ exchanger, regulates temperature-sensitive convulsions and age-related neurodegeneration
Source: bioRxiv. 2026 Feb 3:2025.10.08.681163. Preprint. [Version 2] doi: 10.1101/2025.10.08.681163 (PMC12889452; doi:10.1101/2025.10.08.681163)

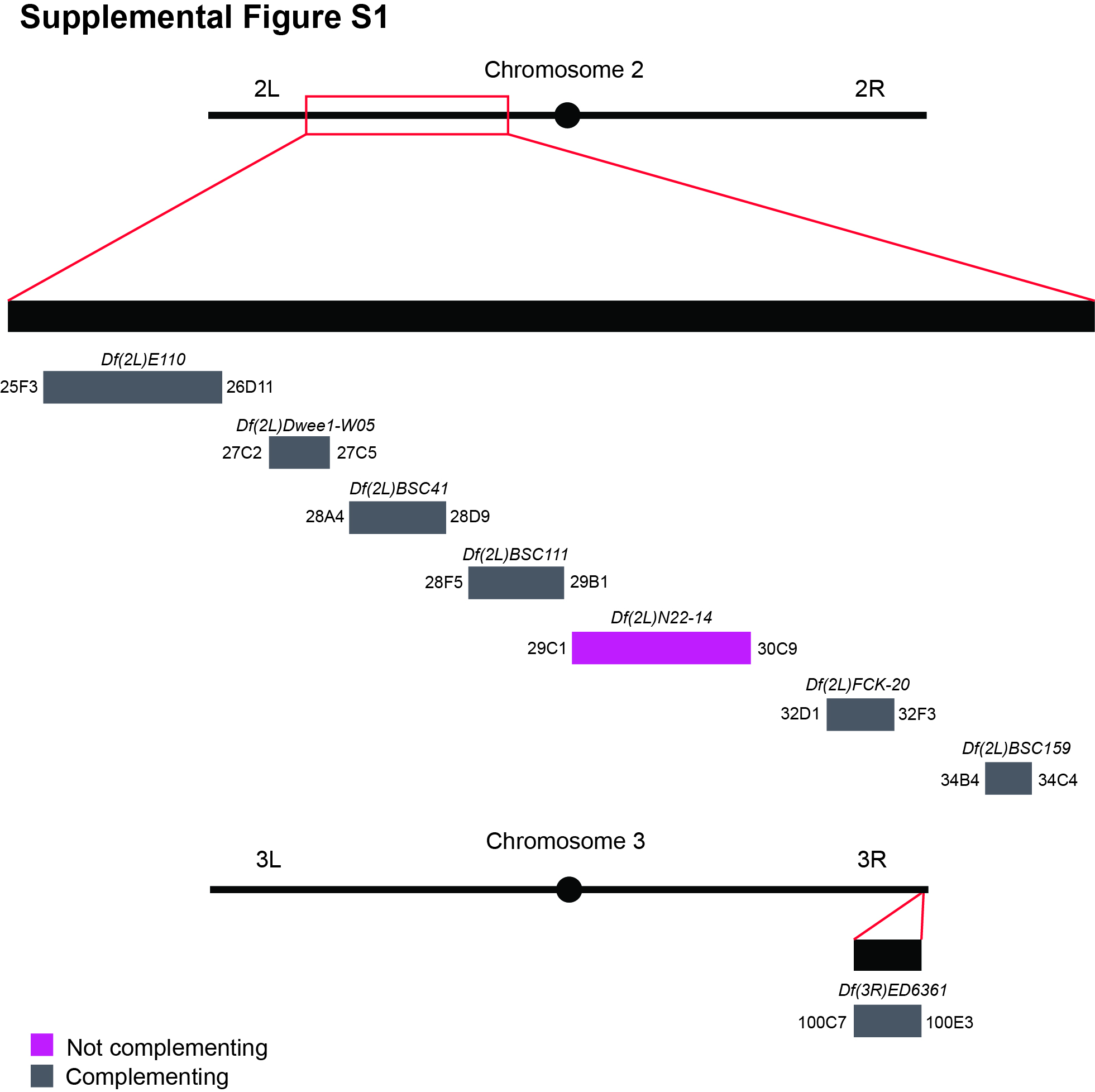

Supplement: Supplement 1 — Schematic of the region of deficiency lines on chromosome 2. 426 mutants fail to complement deficiency lines indicated by the pink boxes. The gray boxes indicate lines that complement the 426 mutant. Deficiency line Df(3R)ED6361 for the right arm of chromosome 3 is used here as a negative control. [file media-1.jpg]

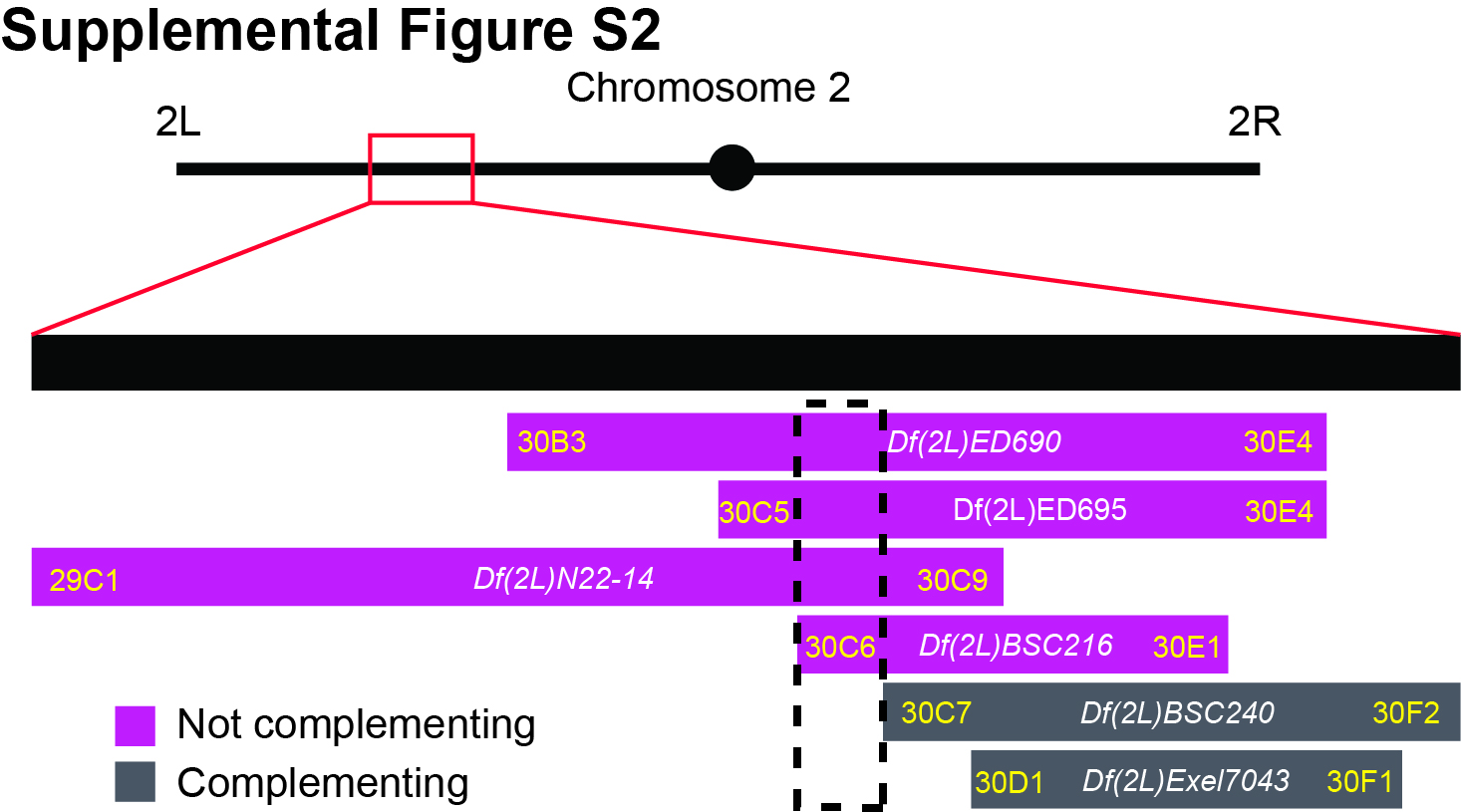

Supplement: Supplement 2 — Schematic of overlapping deficiency lines used for gene mapping. The overlapping region includes the Nckx30c gene locus. 426 mutants fail to complement deficiency lines indicated by the pink boxes, implicating cytological region 30C6–30C7 (black dotted line). The phenotype was scored using a yes/no method where a genotype showing a paralytic phenotype was scored as yes, and the one not showing a phenotype was scored as no. [file media-2.jpg]

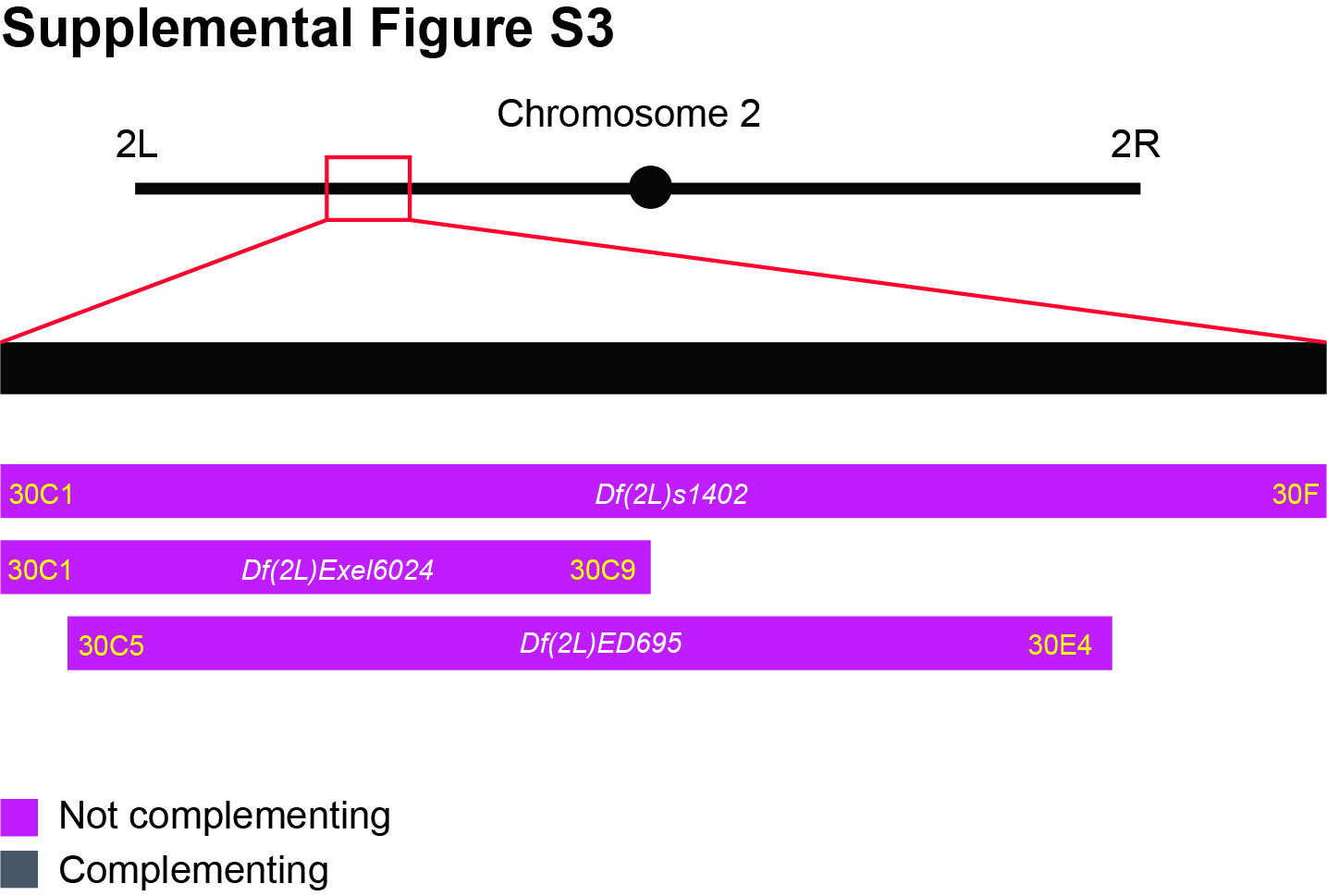

Supplement: Supplement 3 — Schematic of the region of additional deficiency lines on chromosome 2 spanning the region identified as Nckx30c. The pink boxes indicate deficiency lines that failed to complement Nckx30c426. [file media-3.jpg]

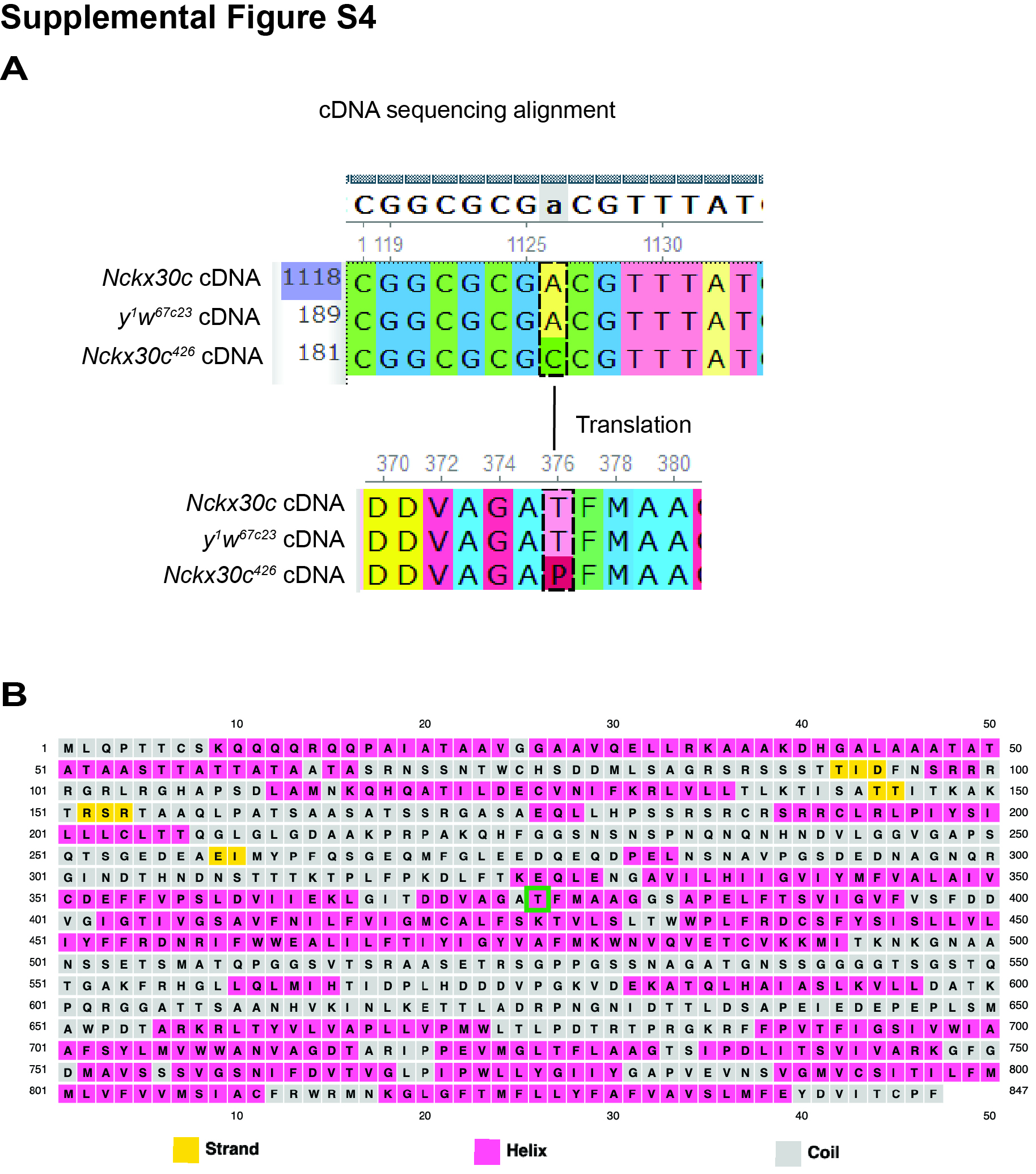

Supplement: Supplement 4 — (A) cDNA sequence of Nckx30c was acquired from database (Flybase.org) and then it was compared with the cDNA sequences from control y1w67c23 and mutant Nckx30c426 flies. The alignment against cDNA from the database shows a change in adenine (A) to cytosine (C) at nucleotide position 1126 in the mutant Nckx30c426, indicated by the black discontinued line. Subsequent translated amino acid sequence alignment shows a change in threonine (T) to proline (P) at residue position 376. (B) Predicted helix region (tool: PSIPRED) shows that the T might be present in the helix region (marked by a green box). Amino acid residues highlighted in yellow represent the beta strand, amino acid residues highlighted in pink represent the alpha helix and amino acid residues highlighted in gray represent irregular coils. [file media-4.jpg]

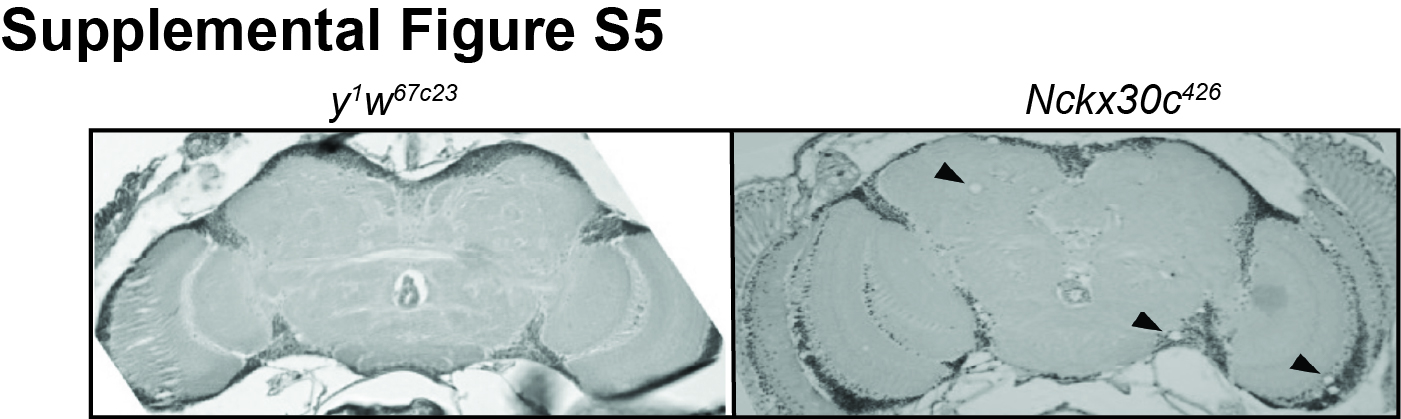

Supplement: Supplement 5 — Representative 5-μm thin paraffin sections at approximately midbrain of 26-day old control y1w67c23 (n=3) and Nckx30c426 (n=3) mutant flies. The presence of holes (arrowheads) is indicative of neurodegeneration. Images were taken at 20x magnification using a light microscope. [file media-5.jpg]

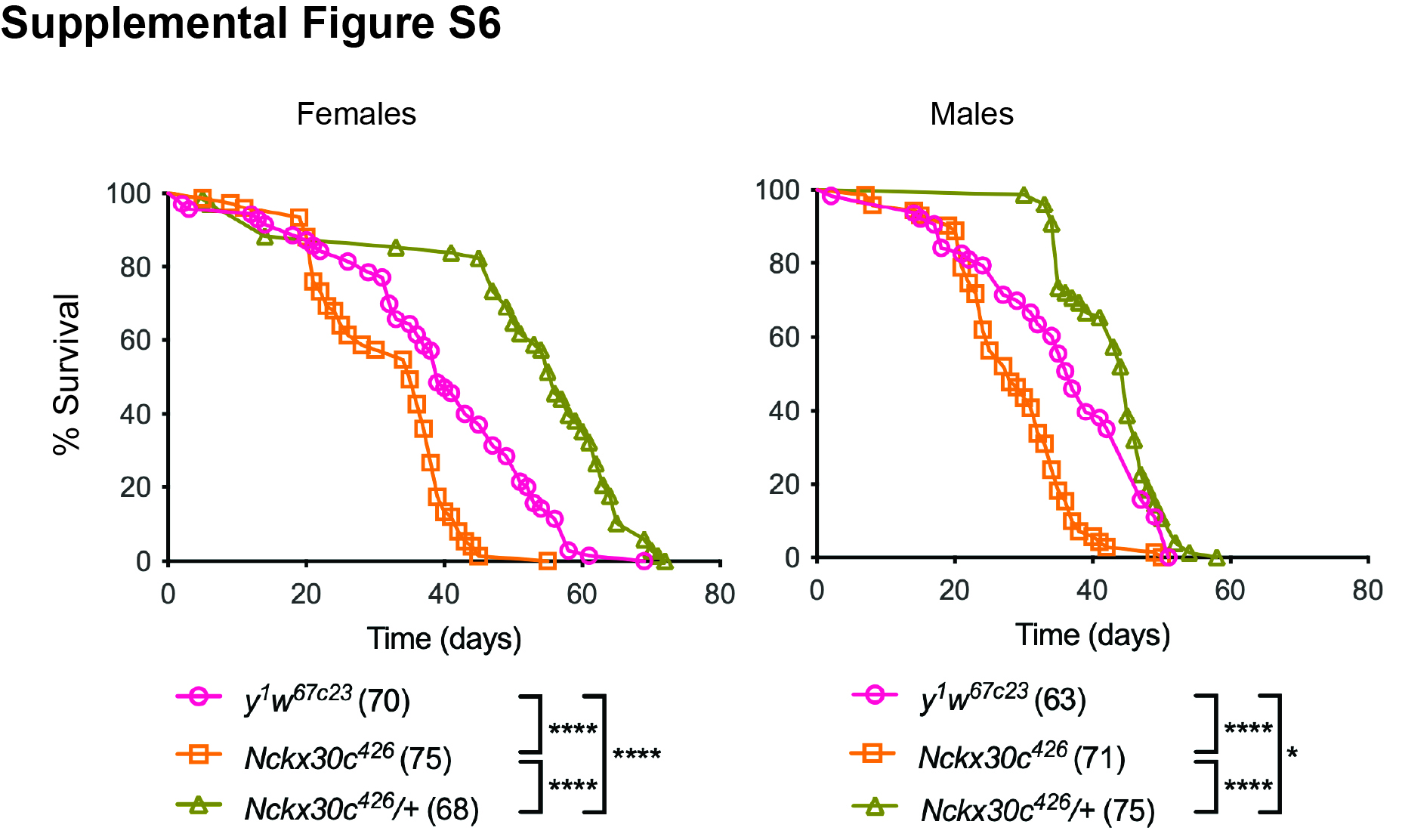

Supplement: Supplement 6 — Lifespan curves of both female and male Nckx30c426mutants, y1w67c23 controls and heterozygous Nckx30c426/+ flies (Nckx30c426 crossed with y1w67c23) at 25°C. Nckx30c426 flies of both sexes display a significantly shorter lifespan in comparison to y1w67c23 and Nckx30c426/+ flies. Numbers in parentheses indicate sample size. Log-Rank (Mantel-Cox); *P ≤ 0.05, ****P ≤ 0.0001. P values are reported in the Supplemental datasheet 1. [file media-6.jpg]

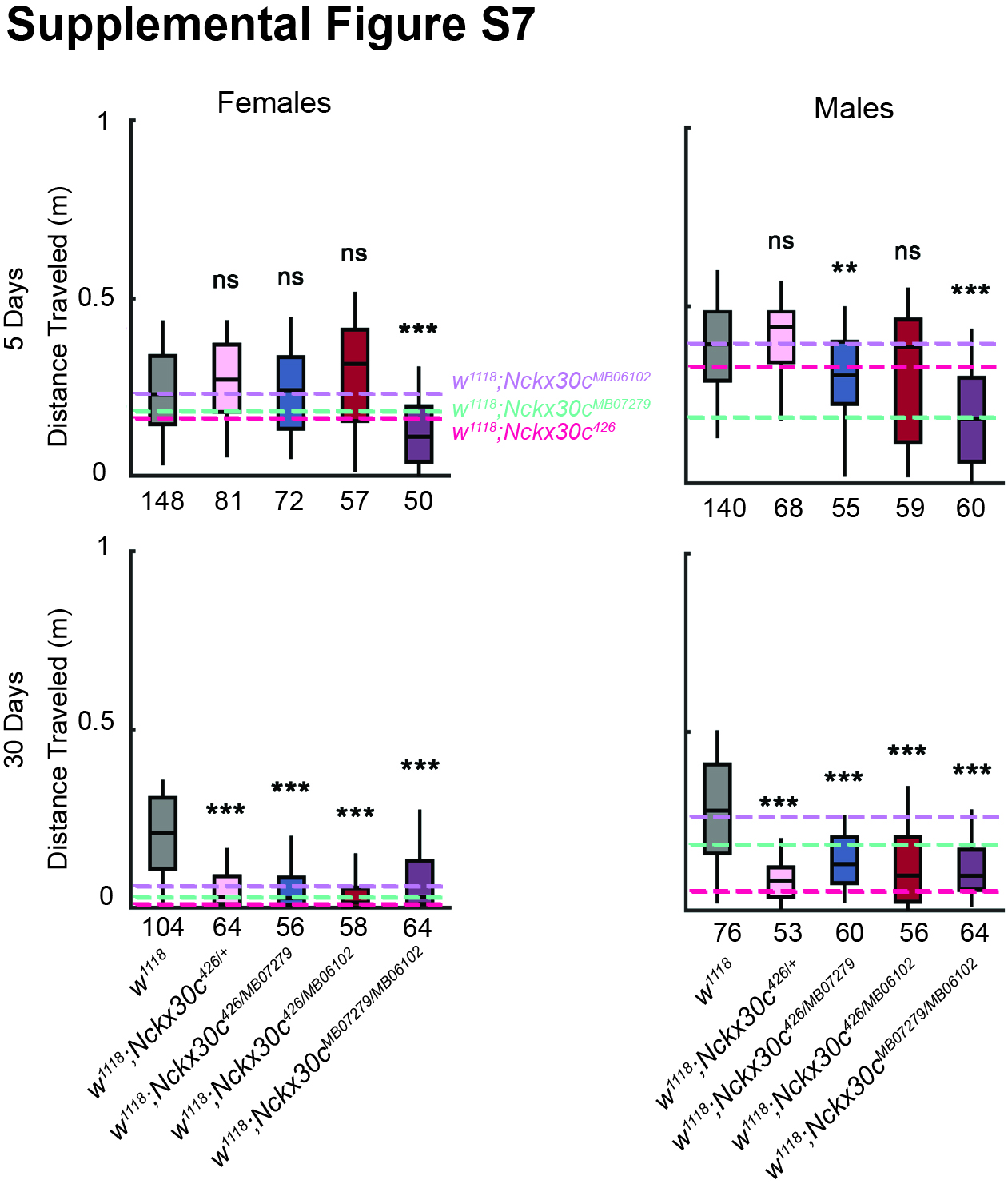

Supplement: Supplement 7 — Automated open-field behavior tracking to measure distance traveled at 22°C in female and male flies at 5 and 30 days of age, respectively. Numbers on the Y axis indicate the sample size of each compound heterozygote. The horizontal lines across each graph indicate the mean distance traveled for the homozygous mutants w1118; Nckx30c426, w1118; Nckx30cMB07279, and w1118; Nckx30cMB06102. A one-way ANOVA (rank-sum post hoc test) was performed to compare each compound heterozygote with w1118; the symbols over each box represent statistical significance. *P ≤ 0.05, **P ≤ 0.01, ***P ≤ 0.001, ns = not significant. P values are reported in the Supplemental datasheet 1. [file media-7.jpg]

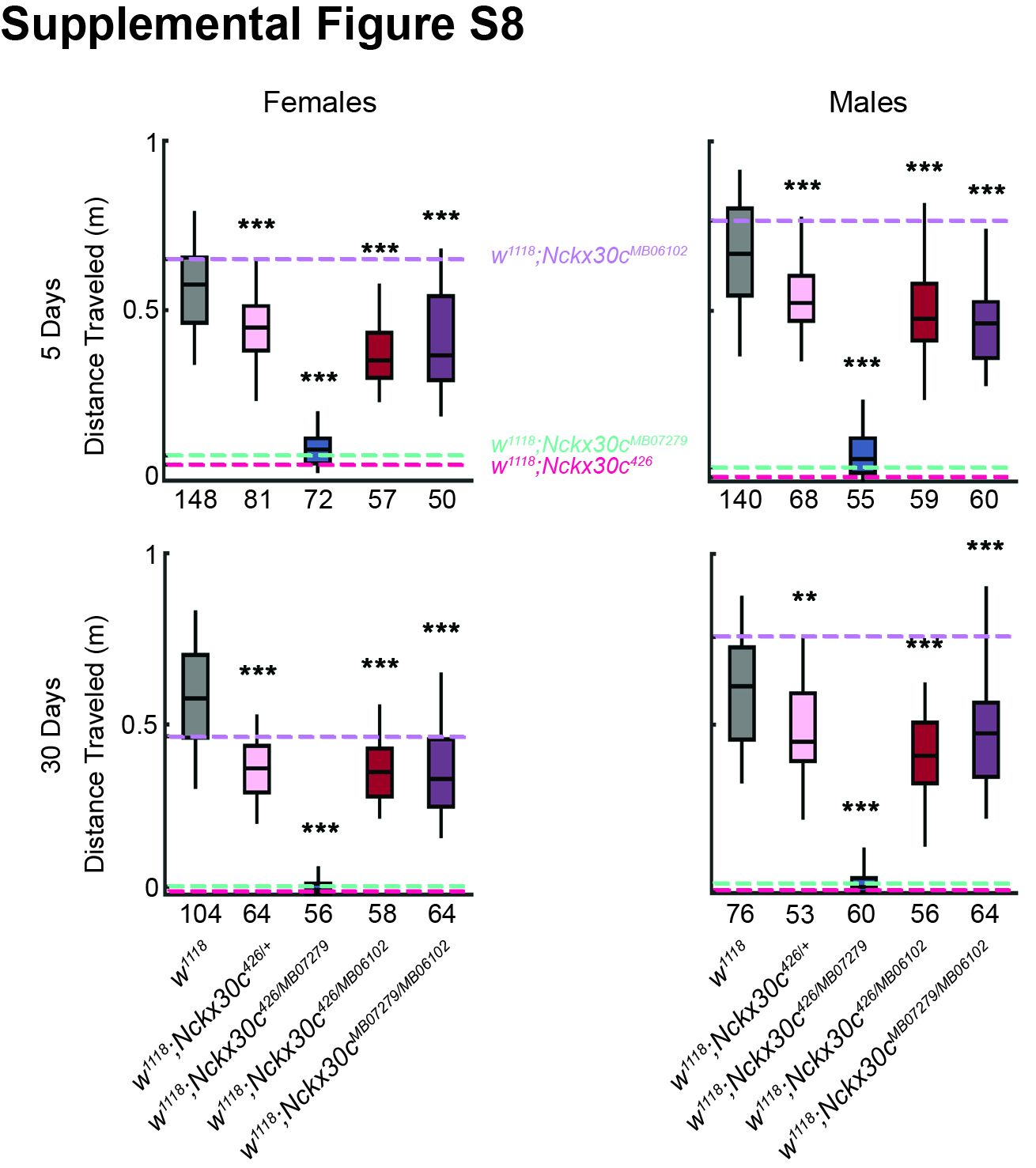

Supplement: Supplement 8 — Automated open-field behavior tracking to measure distance traveled at 39°C in female and male flies at 5 and 30 days of age, respectively. Numbers on the Y axis indicate the sample size of each compound heterozygote. The horizontal lines across each graph indicate the mean distance traveled for the homozygous mutants w1118; Nckx30c426, w1118; Nckx30cMB07279, and w1118; Nckx30cMB06102. A one-way ANOVA (rank-sum post hoc test) was performed to compare each compound heterozygote with w1118; the symbols over each box represent statistical significance. **P ≤ 0.01, ***P ≤ 0.001. P values are reported in the Supplemental datasheet 1. [file media-8.jpg]

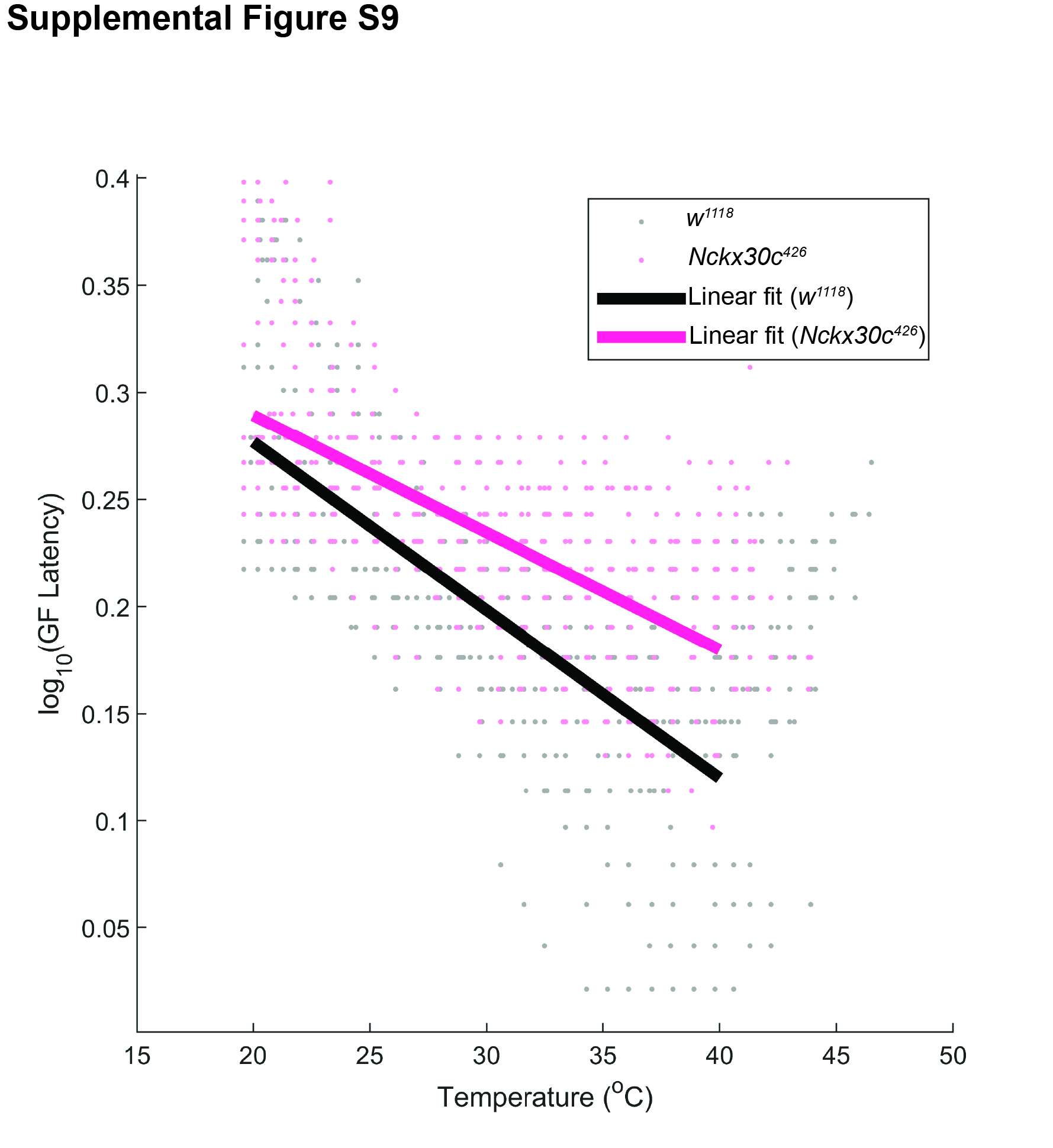

Supplement: Supplement 9 — Scatter plot of the log10 of giant fiber latency as a function of temperature. Lines indicate best fits to the equation: log10(GF latency) = A (ΔT) + B. ΔT indicates the difference from initial temperature (20 °C) and the fit parameter A and B represent the temperature dependence coefficient and latency at initial temperature respectively. For w1118; Nckx30c426: A = −5.48 × 10^−3^ ± 0.26 × 10^−3^, B = 0.29 ± 2.9 × 10^−3^. For w1118: A = −7.84 × 10^−3^ ± 0.28 × 10^−3^, B = 0.28 ± 3.3 × 10^−3^. [file media-9.jpg]

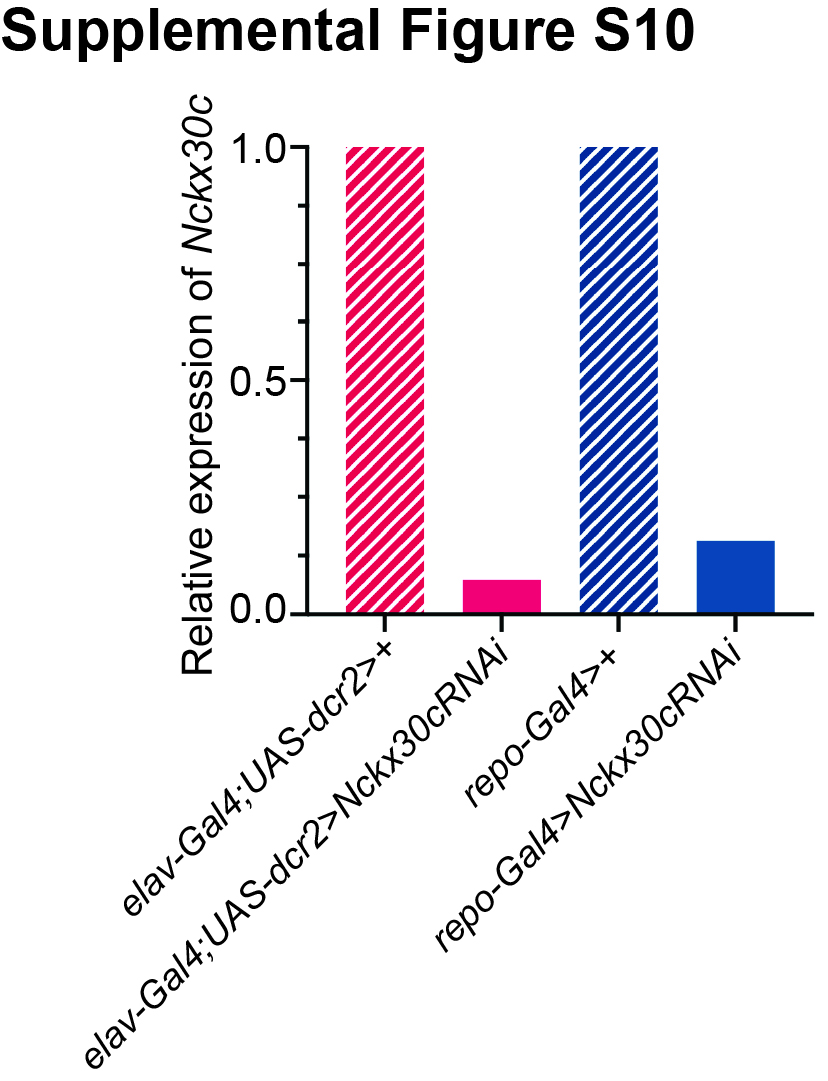

Supplement: Supplement 10 — RT-qPCR analysis of relative expression of Nckx30c gene in heads of elav-Gal4;UAS-dcr2>+, elav-Gal4;UAS-dcr2>UAS-Nckx30cRNAi, repo-Gal4>+ and repo-Gal4>UAS-Nckx30cRNAi flies. Flies with neuronal Nckx30c knockdown (elav-Gal4;UAS-dcr2>UAS-Nckx30cRNAi) show lower expression of Nckx30c than elav-Gal4;UAS-dcr2>+ controls. Flies with glial Nckx30c knockdown (repo-Gal4>UAS-Nckx30cRNAi) also show lower expression of Nckx30c in comparison to repo-Gal4>+ controls. Independent experiment number: n=1. Gene expression data were normalized to the Rp49 gene as an endogenous control, and each knockdown was also normalized to its cell-type control. elav-Gal4;UAS-dcr2>UAS-Nckx30cRNAi was normalized to elav-Gal4;UAS-dcr2>+ and repo-Gal4>UAS-Nckx30cRNAi was normalized to repo-Gal4>+. [file media-10.jpg]

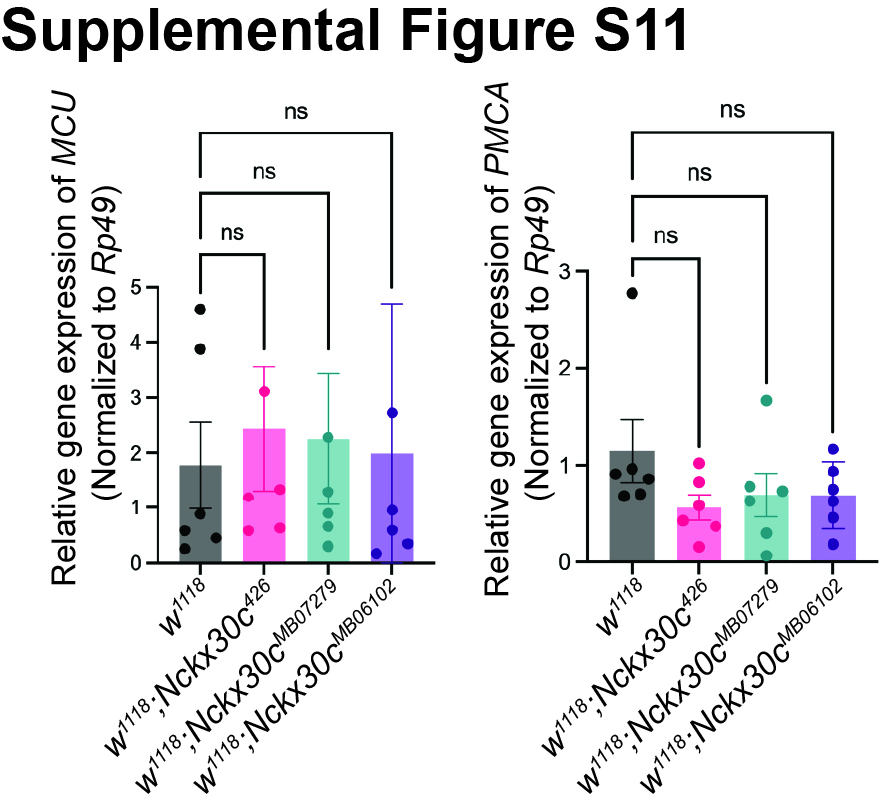

Supplement: Supplement 11 — RT-qPCR analysis of relative expression of MCU and PMCA genes, respectively, in heads of w1118;Nckx30c426, w1118;Nckx30cMB07279 and w1118;Nckx30cMB06102 flies compared to w1118 controls. All samples were from 7-days old male flies. Independent experiment number: n=6. Mean ± SEM; one-way ANOVA with Dunnett’s post-hoc test; ns = not significant. All gene expression data were normalized to Rp49 gene and shown as relative to w1118. P values are reported in the Supplemental datasheet 1. [file media-11.jpg]
